# Serological response to a third booster dose of BNT162b2 COVID‐19 vaccine among seronegative cancer patients

**DOI:** 10.1002/cnr2.1645

**Published:** 2022-06-02

**Authors:** Einat Shacham Shmueli, Yaacov R. Lawrence, Galia Rahav, Amit Itay, Yaniv Lustig, Naama Halpern, Ben Boursi, Ofer Margalit

**Affiliations:** ^1^ Department of Oncology Sheba Medical Center Ramat Gan Israel; ^2^ Sackler Faculty of Medicine Tel‐Aviv University Tel Aviv Israel; ^3^ Department of Radiation Oncology Sidney Kimmel Medical College at Thomas Jefferson University Philadelphia Pennsylvania USA; ^4^ The Infectious Diseases Unit Sheba Medical Center Ramat Gan Israel; ^5^ Central Virology Laboratory Public Health Services, Ministry of Health, Sheba Medical Center Tel Hashomer Israel

**Keywords:** BNT162b2, booster, cancer, COVID‐19

## Abstract

**Background and Aim:**

The BNT162b2 COVID‐19 vaccine (Pfizer/BioNTech), given as a two‐dose series, 3 weeks apart, elicits a serological response in 84–98% of patients with cancer, even if administered while undergoing anticancer treatments. Herein, we report the impact of a third (booster) dose of BNT162b2, delivered 6 months following the second vaccine dose.

**Methods:**

This pilot study included four patients with cancer who were seronegative after two vaccine doses, and received a third (booster) dose of BNT162b2 at 6 months following the second vaccine dose. The four patients received the three vaccine doses between December 2020 and July 2021. Samples were evaluated with an enzyme‐linked immunosorbent assay (ELISA) that detects IgG (Immunoglobulin G) antibodies against the RBD (receptor‐binding domain) of SARS‐CoV‐2.

**Results:**

At a mean time of 19 days (ranges 7–28) after the second vaccination, all four patients were seronegative for RBD‐IgG. However, at a mean time of 21 days (ranges 20–22) after the third dose, three out of the four patients (75%) were now seropositive. Mean RBD‐IgG titers were increased after the third vaccine dose from 0.37 to 2.81 (Student's *t*‐test, *p* = 0.05, two‐sided).

**Conclusions:**

Although limited by the small sample size, our findings suggest that a third (booster) dose administered to patients with cancer, who remain seronegative despite two doses of BNT162b2, may be efficacious in eliciting an antibody response.

## 1 BACKGROUND

The BNT162b2 COVID‐19 vaccine (Pfizer/BioNTech), given as a two‐dose series, 3 weeks apart, elicits a serological response in 84–98% of patients with cancer, even if administered while undergoing anticancer treatments.^1^, ^2^, ^3^ Nonetheless, patients with cancer have lower titers of IgG compared with healthy controls,^1^, ^2^, ^3^ with titers dropping further 4 to 6 months following the second dose.^4^, ^5^ Two recent reports suggest that a third (booster) dose improves the serological response among immunosuppressed transplant patients.^6^, ^7^ Based on these findings, in August 2021, the FDA approved a third dose vaccination for certain immunocompromised individuals. However, the immunogenicity of a third dose vaccination in patients with cancer is unknown.

## 2 METHODS

We previously reported that in an IRB‐approved prospective study in which a two‐dose series of BNT162b2 was administered to patients with cancer receiving active treatment; 18/113 (16%) of patients with cancer remained seronegative after the second vaccine dose.^3^ Here, we report the impact of a third (booster) dose of BNT162b2, delivered 6 months following the second vaccine dose, upon four out of the above‐mentioned 18 seronegative patients. The remaining 14 seronegative patients were either lost to follow‐up or have not received a third vaccine dose and were therefore not included in this pilot study. All patients provided written informed consent.

## 3 RESULTS

The four patients received the three vaccine doses between December 2020 and July 2021. Patient characteristics including cancer diagnosis and treatments are detailed in Table 1. All four patients had concomitant comorbidities: hypertension (1 patient), diabetes (2 patients) and chronic steroid use (4 mg oral dexamethasone, 1 patient). Samples were evaluated with an ELISA that detects IgG antibodies against the RBD (receptor binding domain) of SARS‐CoV‐2. Titers ≥1.1 were defined as positive. At a mean time of 19 days (ranges 7–28) after the second vaccination all patients were seronegative for RBD‐IgG. A confirmatory serum test at mean time of 184 days (ranges 168–206) after the second vaccination showed persistent seronegativity. A third vaccine dose was administered at a mean of 185 days (ranges 168–198) after the second vaccine dose. At a mean time of 21 days (ranges 20–22) after this third dose, three of the patients (75%) became seropositive. Mean RBD‐IgG titers were increased after the third vaccine dose from 0.37 to 2.81 (Student's *t*‐test, *p* = 0.05, two‐sided). All patients continued the same anticancer treatment during the ≥6 months period between the second and third vaccine dose, and none had a documented positive PCR test during this period.

**TABLE 1:** Patient characteristics and serum IgG‐RBD titer after the second and third vaccine doses

	Gender	Age	Comorbidities	Cancer diagnosis	Cancer treatment	RBD‐IgG titer^a^	
						Post 2nd dose	Post 3rd dose
1	Female	46	Chronic steroid treatment	Astrocytoma	Bevacizumab	0.65	3.62
2	Female	73	Diabetes	Pancreatic adenocarcinoma	Gemcitabine	0.26	2.49
3	Male	73	Diabetes	Non‐small cell lung cancer	Pemetrexed	0.26	4.42
4	Male	69	Hypertension	Thymoma	Octreotide	0.30	0.72

a Serum samples were evaluated with an ELISA that detects IgG antibodies against the RBD of SARS‐CoV‐2. ELISA index value below 0.9 was considered as negative, between 0.9 and 1.1 as equivocal, and equal to or above 1.1 as positive.

## 4 CONCLUSIONS

Although limited by the small sample size, our findings suggest that a third (booster) dose administered to patients with cancer who remain seronegative despite two doses of BNT162b2, is efficacious in eliciting an antibody response.

## AUTHOR CONTRIBUTIONS

**Yaacov Lawrence:** Writing – review and editing (equal). **Galia Rahav:** Conceptualization (equal); investigation (equal); project administration (equal); validation (equal); writing – review and editing (equal). **Amit Itay:** Investigation (equal); writing – review and editing (equal). **Yaniv Lustig:** Investigation (equal); writing – review and editing (equal). **Naama Halpern:** Investigation (equal); writing – review and editing (equal). **Ben Boursi:** Investigation (equal); writing – review and editing (equal). **Ofer Margalit:** Conceptualization (equal); data curation (equal); formal analysis (equal); investigation (equal); supervision (equal); writing – original draft (equal); writing – review and editing (equal).

## CONFLICT OF INTEREST

The authors declare that there is no conflict of interest.

## Data Availability

Data sharing is not applicable to this article as no new data were created or analyzed in this study.
